# Supplementary material for: Minimum Displacement in Existing Moment (MDEM)- A new supervised learning algorithm by incrementally constructing the moments of the underlying classes
Source: PLoS One. 2025 Dec 5;20(12):e0336933. doi: 10.1371/journal.pone.0336933 (PMC12680203; doi:10.1371/journal.pone.0336933)
Supplement: Attachment — Submitted filename: review4 plosone_D250618cqxu.pdf [file pone.0336933.s001.pdf]

Report on "Minimum Displacement in Existing Moment (MDEM)- A new supervised learning algorithm by incrementally constructing the moments of the underlying classes" by A. M. Nizam

C Xu

June 18, 2025

1 General Assessment

In this paper, the author proposes a supervised learning algorithm based upon Minimum Displacement in Existing Moment (MDEM). By some numerical experiments, the author evaluates the performances of MDEM in mean, variance, 3th and 4th central moment and obtain accuracy scores 73.43

By the comparison, the author asserts that the MDEM in mean runs better than KNN with 3, 5, 7 neighbors and 8 (eight) of the NN models under consideration in 2-fold cross validation technique, and that the best algorithm under 2-fold cross validation should be SVM, which has a run time accuracy of 76.63

The manuscript presents a novel supervised learning algorithm called Minimum Displacement in Existing Moment (MDEM) that incrementally constructs class moments. The work demonstrates solid theoretical foundations and comprehensive empirical validation using the Pima Indian Diabetes dataset. While the concept is something new and well-presented, several aspects require clarification and improvement before publication.

2 Strengths

- **Novel Algorithm**: The proposed MDEM approach offers a unique perspective on classification by minimizing moment displacement, which hasn't been extensively explored in literature.

- **Computational Efficiency**: The paper convincingly demonstrates the algorithm's constant-time classification complexity (O(kd)), which is a significant improvement over KNN's linear complexity.

- **Comprehensive Evaluation**: The comparison with five state-of-the-art algorithms (NN, SVM, RF, KNN, LR) using both k-fold and stratified k-fold validation provides robust evidence of performance.

- **Theoretical Foundation**: The mathematical derivations of raw and central moments (Section 2) are technically sound and well-presented.

3 Concerns and Suggestions

3.1 Methodological Clarifications

- The manuscript would benefit from clearer pseudocode formatting in Algorithms 1-4. Consider using standard algorithmic packages like `algorithm2e` for better readability.

- The moment calculation formulas (Equations 1-4) are correct but could be more clearly connected to the algorithmic implementation. A small numerical example showing moment calculation would be helpful.

3.2 Empirical Evaluation

- The performance range of 83.19%-95.82% of the best algorithm is notable, but the paper should discuss why higher-order moments (3rd, 4th) underperform compared to mean and variance.

- The comparison with neural networks mentions performance differences based on layers/nodes, but doesn't explain why MDEM outperforms smaller NNs while being outperformed by larger ones. This deserves deeper analysis.

- The results tables (Tables 2-3) would be more impactful with statistical significance indicators (e.g., p-values or confidence intervals).

3.3 Presentation, Organization, and Technical Corrections

- The abstract could better highlight the key innovation - the incremental moment updating mechanism that enables constant-time classification.
- Figure 1 effectively illustrates the class cardinality weighting concept. Figures 2-3 showing performance comparisons could be improved with proper axis labels and clearer differentiation between MDEM variants.
- The conclusion should more explicitly state the practical scenarios where MDEM would be preferred over other algorithms, given its trade-off between accuracy and speed.
- The time complexity analysis in Section 4 is thorough but could benefit from Big-O notation consistency (sometimes uses ?, sometimes omits).
- References should be formatted consistently according to journal guidelines (currently mixing journal abbreviations and full names).

4 Recommendation

Recommend **Major Revision** before acceptance. The work presents a valuable contribution to supervised learning with its novel moment-based approach, but requires:

1. Clearer explanations of why higher-order moments underperform.
2. More rigorous statistical analysis of results.
3. Improved visualization of performance comparisons
4. Better positioning of the algorithm's practical applicability

The manuscript has strong potential for publication in PLOS ONE after addressing these concerns. The proposed method offers a computationally efficient alternative to existing algorithms, particularly in scenarios requiring

rapid classification of streaming data where slight accuracy reductions are acceptable.
