# Supplementary material for: Minimum Displacement in Existing Moment (MDEM)- A new supervised learning algorithm by incrementally constructing the moments of the underlying classes
Source: PLoS One. 2025 Dec 5;20(12):e0336933. doi: 10.1371/journal.pone.0336933 (PMC12680203; doi:10.1371/journal.pone.0336933)
Supplement: Attachment — Submitted filename: GuoningYu_comments.pdf [file pone.0336933.s002.pdf]

Review Report

Guoning Yu

June 20, 2025

Manuscript Information

Title: Minimum Displacement in Existing Moment (MDEM) - A new supervised learning algorithm by incrementally constructing the moments of the underlying classes

Journal: PLOS ONE

Manuscript ID: PONE-D-25-26429

Summary

This manuscript presents a novel supervised learning algorithm for classification called Minimum Displacement in Existing Moment (MDEM). The author claims that the performances of MDEM involving lower order moments are comparable to those of existing state-of-the-art classification methods.

The algorithm the author proposes can be seen as a variation of the nearest centroid classifier (or Rocchio algorithm). Instead of calculating the distance between the test data point to the mean of each class, the proposed algorithm calculates the displacement between the original mean of each class and the mean after assigning a test data point to each class, and then pick the class with the least displacement to actually assign the test data point. Note that the statistic *mean* can be replaced by other statistics as the author has stated.

The manuscript is well-structured and provides a clear explanation of the algorithm, and experimental results. However, there are aspects that require clarification and improvement. My main concern is the lack of theoretical justification for the algorithm, particularly regarding the technique of multiplication by the cardinality of the class when calculating the temporary mean (or other statistics), e.g., line 10 in Algorithm 2.

Overall, the manuscript is a good contribution to the field of machine learning and provides a new perspective on classification algorithms. I recommend publication and that the author can address the following major and minor comments.

Major Comments

1. Even though the algorithm proposed makes sense intuitively and the author provides experimental results with specific data set, I would recommend the author to provide

a theoretical justification for the algorithm. For example, it does seem necessary to multiply the temporary mean by the cardinality of the class when calculating the displacement, but it is not clear if this is sufficient to account for the effect that *the class with high cardinality eat up every new point due to its gravitational pull* (manuscript line 166). The same concern applies to the use of other moments.

2. I recommend the author to include more related methods in the introduction section, such as the nearest centroid classifier and clarify if there is any other similar methods that have been proposed in the literature. This will help to provide a clearer context for the proposed algorithm and its contributions.

3. The algorithm has only been tested on one specific data set, the PID data set. I would recommend the author to test the algorithm on other data sets and compare the performances with other state-of-the-art classification methods. This will help to validate the effectiveness and generalizability of the proposed algorithm.

Minor Comments

This should not be a complete list since I have not read the manuscript in very great detail, but I will provide some minor comments that I have noticed so far.

1. It is better to specify that the algorithm is a supervised learning algorithm for **classification** in the abstract. Same applies to the introduction.

2. The first sentence of the abstract can be rephrased like: *We introduce a supervised learning method that classifies each test point by selecting the class for which its inclusion causes minimum displacement of the class's existing n-th central moment.*

3. Manuscript line 36: $O(logn)$ should be $O(\log n)$.

4. Every citation mark for the whole manuscript has a space before it, e.g., manuscript line 35, 37, 38, etc.

5. Manuscript line 100: move the commas before both "or"

6. Manuscript line 112-122: remove the colons after all "section", also remove the colons after "Algorithm" and "Fig" in later sections.

7. Manuscript line 142: the attributes should be all numerical according to the context, which is better to be specified.

8. Manuscript line 192: *after the training phase, we have the* <u>*n-th central moment*</u> *of each class...* It should not be *mean*.

9. Manuscript line 197: should be *the number* <u>*of*</u> *attributes*
