# Supplementary material for: Minimum Displacement in Existing Moment (MDEM)- A new supervised learning algorithm by incrementally constructing the moments of the underlying classes
Source: PLoS One. 2025 Dec 5;20(12):e0336933. doi: 10.1371/journal.pone.0336933 (PMC12680203; doi:10.1371/journal.pone.0336933)
Supplement: Attachment — Submitted filename: Response-To-Reveiwers.pdf [file pone.0336933.s003.pdf]

Response to reviewers

Ahmed Mehedi Nizam

August 8, 2025

1 Reviewer-1

1.1 Comment-I.1

Reviewer: 1
I) Insufficient clarity in expressing core concepts

- The paper proposes classifying data points into the category with the minimum moment displacement, but it lacks an intuitive explanation of the physical meaning of "moment displacement" and why this index can effectively characterize the correlation between data points and categories. For example, it only states that Euclidean distance is calculated and weighted by the category cardinality, but does not clarify the theoretical basis of the weighting strategy (e.g., why cardinality balances the "attractiveness" of categories), making it difficult for readers to understand the rationality of the decision-making mechanism.

Author:
We will attain the reviewer's concern in the following two ways:

1. Now, we have provided a more detailed intuitive explanation of the physical meaning of "moment displacement" in section 2 of the revised manuscript. To be precise, in section 2, now we have explicitly mentioned that the 3rd and 4th central moment are non-normalized skewness and kurtosis of the distribution. There were no physical interpretations of the 3rd and 4th order moments in the previous version of the manuscript. Now, we have added it. Moreover, in the previous as well as the current version of the manuscript, we have mentioned that the first raw moment and second central moment as used in the analysis are mean and variance of the distribution respectively. So, whenever we say "moment displacement", we seek to apply some sort of "Minimum Perturbation Criteria", i.e., insertion of new data point into the class that is least perturbed due to such inclusion in terms of its existing mean, variance, skewness and kurtosis,

(which one is applicable). It is to be mentioned in this regard that "Minimum Perturbation Criterion" is widely used in machine learning as well as in other disciplines.

2. To justify the weighting strategy, we have added a whole new section titled "MDEM as a size-weighted minimum perturbation classifier", which establishes MDEM algorithm as a size-weighted minimum perturbation classifier. To justify the weighting strategy of the displacement in moments by the respective class cardinality, here we have given a formal proof of the fact that the displacement in the n-th central moment and displacement in 1st raw moment (mean) of a class due to inclusion of a new instance, are inversely proportional to the respective class cardinality. So, to obtain an unbiased measure of displacement metric, we need to multiply it by the respective class cardinality. The formal proof is given in section 5 of the revised manuscript.

1.2 Comment-I.2

Reviewer: 1
The algorithm discusses the applications of the mean (first-order raw moment) and second- to fourth-order central moments, but does not explain why these orders are chosen. Additionally, whether increasing the order will continuously improve classification performance and its boundary conditions are not clearly stated.

Author:
The first raw moment as well as 2nd, 3rd and 4th central moments are mean, variance, skewness and kurtosis of the distribution, each of which have specific statistical interpretations. Moments, higher in order than the 4th order moments, are known as hyper-skewness (odd ones) and hyper-kurtosis (even one). These higher order moments are rarely used in statistical analysis and only capture fine-details of skewness and kurtosis respectively. We have added the following paragraph in the 'Methodology' section of our manuscript to address the issue:
"Here, we consider only first raw moment as well as 2nd, 3rd and 4th order central moments as they capture some specific attributes of the underlying distribution, namely, mean, variance, skewness and kurtosis respectively. It is to be noted in this regard that moments, higher in order than the 4th order moments, are known as hyper-skewness (odd ones) and hyper-kurtosis (even one) that are rarely used in statistical analysis and only capture fine details of skewness and kurtosis respectively."
Moreover, whether the performance of MDEM algorithms increases or decreases with the order of moments, depends on the underlying dataset used in the analysis. If the dataset favors lower order moments over higher order ones, then using lower order moments delivers better performance and, in our cases, we have reported exactly this incident. However, there may be other datasets that

prefer homogeneity in skewness and kurtosis to that in mean and variance and, for those datasets, MDEM involving higher order moments may perform better - We have added these reasonings in the 'Discussion' subsection under 'Results' section of our revised manuscript.

1.3 Comment II

Reviewer: 1
Limitations in time complexity analysis

1. The paper only theoretically deduces that the training time complexity is O(P) and the testing time is constant, but does not consider the actual overhead of calculating power sums for high-dimensional data (when d is large) or high-order moments (when n is large). For example, when n=4, each data point needs to calculate the 4th power four times. When n and d are large, the time complexity may deviate from the theoretical expectation, and the discussion of boundary conditions is lacking.

2. When comparing with the O(nd) testing complexity of KNN, the paper does not mention the moment calculation cost of MDEM in processing high-dimensional data (e.g., Euclidean distance calculation involves N-dimensional vectors), which may lead to an incomplete understanding of the algorithm's efficiency advantages among readers.

Author:

1. In section: 4.1 (Time complexity of MDEM in mean) of our manuscript, we have mentioned that the initial training phase time complexity of MDEM in mean is actually $O(N \times P)$, where P is the number of training rows and N is the number of attributes. Then, we discuss the issue for a specific problem. For a specific problem, the number of attributes, N, does not change. So, as we have discussed in section: 4.1., the time complexity reduces to simply $O(P)$ for a single problem under consideration.

   Moreover, in section of 4.1 (Time complexity of MDEM in mean), we have also discussed that the training phase time complexity of MDEM in mean is actually $O(M \times N)$, where M is the number of classes and N is the number of attributes. M and N are fixed for a specific problem. So, if we confine ourselves to a specific problem, then the testing phase time complexity is constant. But, in general the time complexity is still $O(M \times N)$ as a whole as we discussed in section: 4.1.

   Additionally, in section: 4.2 (Time complexity of MDEM in n-th central moment), we mention that the initial training phase time complexity of MDEM in n-th central moment is actually $O(n \times N \times P)$, where n is the number of moments, N is the number of attributes and P is the number of training instances. For a specific problem the value of N is fixed and for

a specific implementation of MDEM algorithm, n is fixed, which reduces the time complexity to $O(P)$. However, as we have discussed in section: 4.2., the original time complexity remains at $O(n \times N \times P)$. Moreover, as we have discussed in section: 4.2., the testing phase time complexity of the algorithm is $O(n \times N \times M)$, where n is the number of moment, N is the number of attributes and M is the number of classes. For a specific problem, the value of N and M are fixed and for a specific implementation of MDEM algorithm, n is fixed, which reduces the time complexity to a constant, i.e., it does not depend upon any variable and is therefore fixed for every new testing instance. However, as we have discussed in section: 4.2, the actual testing phase time complexity stays at $O(n \times N \times M)$.

2. In section: 4.1, we have discussed that the testing phase time complexity of MDEM in mean is actually $O(N \times M)$, where N is the number of attributes and M is the number of classes. Moreover, in section: 4.2., we have described the testing phase time complexity of MDEM in n-th central moment is $O(n \times N \times M)$, where n is the number of moment, N is the number of attributes and M is the number of classes. We think, explanations provided in section: 4.1 and 4.2, should resolve any such ambiguity.

1.4 Comment-III

Reviewer: 1 III. Inadequacies in experimental design

1. It only verifies the performance of the mean, variance (second-order central moment), third-order, and fourth-order central moments, but does not set up control groups (such as combinations of mixed-order moments), making it impossible to prove the optimality of a single-order moment or explore the correlation between the order and data features (e.g., whether certain features are more suitable for high-order moments).

2. The paper emphasizes that the model can be updated incrementally, but the experiment does not compare the performance differences between "dynamic update" and "static model" (i.e., not updating moments after training), making it impossible to prove the practical value of this innovation.

3. The comparison metrics only use a single accuracy index, which is difficult to comprehensively measure the model's performance in imbalanced samples. It is recommended to add common machine learning metrics such as F1-score, Precision, Recall, and MCC to comprehensively evaluate the algorithm's performance under multiple metrics.

4. All experiments only use one dataset (PID). The PID dataset used in the experiment has fewer feature dimensions (8 dimensions) and a relatively sufficient sample size (768 samples), making it difficult to prove the algorithm's wide applicability. For example: How does the algorithm perform

when facing high-dimensional data? Additionally, how does the algorithm perform on small-sample datasets? Adding these comparisons can further corroborate the algorithm's superiority.

Author:

1. The reviewer has rightly pointed out that we do not go for checking optimality of single order moments. However, as we have mentioned in our revised manuscript that MDEM is designed for speed and not for optimality. It performs with greater speed delivering only sub-optimal performances. In our revised manuscript, we have added a subsection titled 'Discussion' under result section in this regard, which reads as follows.

   "MDEM algorithms are better suited in numerous situations, where speed in running time is more important than cutting-edge accuracy. For example, MDEM algorithms can be practically deployed to different text classification scenarios, e.g., classifying documents according to search query, email filtering (spam vs non-spam and also categorization and labelling of emails), categorization of news articles (sports, politics, international etc.), populating newsfeeds for users in social media, sentiment analysis (classifying reviews as positive, negative and neutral), support ticket routing (assign incoming tickets to the right department), legal and medical documents tagging (tag documents based on predefined categories) and the alike. These are some of the applications, where speed is preferred to optimality as the data here are massive in quantity and consequences of possible misclassification are relatively less dangerous."

   In addition, we have also discussed that the performance of MDEM algorithms may vary depending upon the underlying dataset. If the dataset prefers mean and variances over higher order moments like skewness and kurtosis, then MDEM involving lower order moments will perform well. However, if the dataset prefers skewness and kurtosis over lower order moments, then MDEM with higher order moments will perform well. We have discussed the issue in the 1st paragraph newly added "Discussion" subsection in "Results" section of our revised manuscript, that reads as follows.

   "However, which version of MDEM algorithm will perform better than the others depends mostly on the underlying dataset used in the analysis. If the dataset favors lower order moments like mean and variance over higher order ones like skewness and kurtosis, then using lower order moments delivers better performance and, in our cases, we have reported exactly the same incident. However, there may be other datasets that prefer homogeneity in skewness and kurtosis to that in mean and variance and, for those datasets, MDEMs involving higher order moments may perform better."

2. As the model can effectively train itself in constant time with a simple assignment of temporary moment to the new moment, we think, we should go for the 'dynamic one' as it learns continuously without incurring much overhead.

3. To address the reviewer's concern, we have now added F1-score, Precision, Recall, and MCC in our analysis of the performance of MDEM algorithms.

4. We have now extended our analysis using Wisconsin breast cancer dataset, which has 30 features and 569 instances. We think this will address the reviewer's concerns involving high-dimensional and relatively small sample datasets. Results for Wisconsin breast cancer dataset are even better than that of PID dataset as we have discussed the issue in the revised manuscript.

2 Reviewer-2: C Xu

2.1 Comment-1

Reviewer-2: C Xu: Methodological Clarifications

1. The manuscript would benefit from clearer pseudocode formatting in Algorithms 1-4. Consider using standard algorithmic packages like algorithm2e for better readability.

2. The moment calculation formulas (Equations 1-4) are correct but could be more clearly connected to the algorithmic implementation. A small numerical example showing moment calculation would be helpful.

Author

1. As per reviewer's suggestion, we have now reformatted algorithm: 1-4 using algorithm2e package.

2. Following reviewer's suggestion, a numerical example of moment calculation has been embedded at the beginning of section: 3.2 (MDEM in n-th central moment).

2.2 Comment-2

Reviewer-2: C Xu: Empirical Evaluation

1. The performance range of 83.19%-95.82% of the best algorithm is notable, but the paper should discuss why higher-order moments (3rd, 4th) underperform compared to mean and variance.

2. The comparison with neural networks mentions performance differences based on layers/nodes, but doesn't explain why MDEM outperforms smaller NNs while being outperformed by larger ones. This deserves deeper analysis.

3. The results tables (Tables 2-3) would be more impactful with statistical significance indicators (e.g., p-values or confidence intervals).

Author:

1. Following the reviewer's suggestion, we have added a 'Discussion' subsection under 'Results' section in our manuscript that discusses the issue. It reads as follows.

   "From the above discussion, it can be seen that the MDEM algorithm performs within the [83.19% − 95.82%] bound of the best algorithm under consideration for PID dataset. Moreover, for Wisconsin breast cancer dataset, its performance seems to swing between [88.85% − 96.41%]. Moreover, for both the datasets, MDEMs involving lower order moments perform remarkably well as compared to MDEMs involving higher order moments. In fact, the lower bounds of the afore-mentioned accuracy scores are attributed to MDEMs involving 3rd and 4th order central moments, while the upper bounds are practically due to MDEMs in mean. Also, as we can be seen from Table: [5], [8], [11] and [14], MDEMs in mean and variance have consistently obtained better F1 score and MCC as compared to MDEMs in skewness and kurtosis. However, which version of MDEM algorithm will perform better than the others depends mostly on the underlying dataset used in the analysis. If the dataset favors lower order moments like mean and variance over higher order ones like skewness and kurtosis, then using lower order moments delivers better performance and, in our cases, we have reported exactly the same incident. However, there may be other datasets that prefer homogeneity in skewness and kurtosis to that in mean and variance and, for those datasets, MDEMs involving higher order moments may perform better."

2. The reviewer has rightly pointed out that MDEM outperforms smaller NNs, while being outperformed by larger ones in case of PID dataset. The query can be equivalently reframed as "Why larger NNs outperformed smaller ones in PID dataset". This is a valid question and requires deeper attention indeed. However, from our point of view, the answer to this query may go beyond the scope of the current study, as the study mainly focuses on a new supervised learning algorithm, e.g., MDEM. To answer this question, we may need to revisit NN architectures involving layers, nodes, batch and epoch size, activation function etc. Moreover, apart from NN architecture, the performance issue may be attributed to the idiosyncrasy of the PID dataset. We think, these issues will drag us beyond the scope of the current study.

3. Apart from the accuracy scores, we have now reported precision, recall, F1 score and MCC of different algorithms in our revised manuscript. Instead of just 02 tables for accuracy scores in our previous manuscript, we have now reported the entire results for PID dataset that includes accuracy scores, precision, recall, F1 score and MCC in 06 separate tables.

Moreover, we have now extended the study using Wisconsin breast cancer dataset, which demands for another 06 tables. We think, all these things have made the statistical testing more robust than it was before and serves reviewer's query in this regard.

2.3 Comment-3

Reviewer-2: C Xu: Presentation, Organization, and Technical Corrections

1. The abstract could better highlight the key innovation - the incremental moment updating mechanism that enables constant-time classification.
2. Figure 1 effectively illustrates the class cardinality weighting concept. Figures 2-3 showing performance comparisons could be improved with proper axis labels and clearer differentiation between MDEM variants.
3. The conclusion should more explicitly state the practical scenarios, where MDEM would be preferred over other algorithms, given its tradeoff between accuracy and speed.
4. The time complexity analysis in Section 4 is thorough but could benefit from Big-O notation consistency (sometimes uses ?, sometimes omits).
5. References should be formatted consistently according to journal guidelines (currently mixing journal abbreviations and full names)

Author:

1. Following reviewer's suggestion, we have now added the following line in the abstract: "After each such inclusion, the n-th central moment of the corresponding class is updated by some incremental calculations in constant time, i.e., each class evolves gradually and changes its definition incrementally after the inclusion of every new data point."
2. Following the reviewer' suggestion, we have now edited Figure: 2 & 3 showing proper axis labels. Moreover, each of the column/bar in Figure: 2 & 3, now displays the numeric values, which adds more clarity to the diagram. In addition, we have added Figure: 4 & 5, which presents the results for the newly included Wisconsin breast cancer dataset.
3. To address the reviewer's concern, we have now added the following line in the conclusion: "So, whenever we are in need of a multiclass learning algorithm that is intended to handle massive amount of data e.g., newsfeed generation in social media, email filtering, text classification and categorization, support ticket routing, sentiment analysis, different variants of our proposed MDEM algorithms can come up as a suitable choice with a far better running time and a slightly less than optimal performance."
4. Following the reviewer's suggestion, we have now consistently used Big-O notation throughout the manuscript.

5. Following the reviewer' suggestion, we have now reformatted the bibliography according to journal requirements.

2.4 Comment-4

Reviewer-2: C Xu: Recommendation

1. Clearer explanations of why higher-order moments underperform.
2. More rigorous statistical analysis of results.
3. Improved visualization of performance comparisons
4. Better positioning of the algorithm's practical applicability

Author:

1. We have now added a clearer explanation of why higher-order moments underperform for the datasets used in the analysis in the newly added 'Discussion' subsection of the manuscript, which reads as follows.

   "From the above discussion, it can be seen that the MDEM algorithm performs within the [83.19% – 95.82%] bound of the best algorithm under consideration for PID dataset. Moreover, for Wisconsin breast cancer dataset, its performance seems to swing between [88.85% – 96.41%]. Moreover, for both the datasets, MDEMs involving lower order moments perform remarkably well as compared to MDEMs involving higher order moments. In fact, the lower bounds of the afore-mentioned accuracy scores are attributed to MDEMs involving 3rd and 4th order central moments, while the upper bounds are practically due to MDEMs in mean. Also, as we can be seen from Table: [5], [8], [11] and [14], MDEMs in mean and variance have consistently obtained better F1 score and MCC as compared to MDEMs in skewness and kurtosis. However, which version of MDEM algorithm will perform better than the others depends mostly on the underlying dataset used in the analysis. If the dataset favors lower order moments like mean and variance over higher order ones like skewness and kurtosis, then using lower order moments delivers better performance and, in our cases, we have reported exactly the same incident. However, there may be other datasets that prefer homogeneity in skewness and kurtosis to that in mean and variance and, for those datasets, MDEMs involving higher order moments may perform better."

2. Following reviewer's suggestions, in addition to reporting the accuracy scores, we have now added precision, recall, F1 score and MCC for each of the algorithms, which substantially increased the volume of the manuscript. Moreover, we have now reported the performance of all 19 algorithms for Wisconsin breast cancer dataset as well.

3. Figure: 2 & 3 have been modified according to reviewer's suggestions. Data label over each column/bas is added for better visualization. Moreover, Figure: 4 & 5 have been newly added that visually represent the results of different algorithm using another new dataset, namely, Wisconsin breast cancer dataset.

4. To address the issue, we have now added a separate paragraph under the newly appended 'Discussion' subsection of our revised manuscript, which reads as follows. "MDEM algorithms are better suited in numerous situations, where speed in running time is more important than cutting-edge accuracy. For example, MDEM algorithms can be practically deployed to different text classification scenarios, e.g., classifying documents according to search query, email filtering (spam vs non-spam and also categorization and labelling of emails), categorization of news articles (sports, politics, international etc.), populating newsfeeds for users in social media, sentiment analysis (classifying reviews as positive, negative and neutral), support ticket routing (assign incoming tickets to the right department), legal and medical documents tagging (tag documents based on predefined categories) and the alike. These are some of the applications, where speed is preferred to optimality as the data here are massive in quantity and consequences of possible misclassification are relatively less dangerous."

3 Reviewer-3: Guoning Yu

3.1 Major comments

Guoning Yu (Reviewer-3)

1. Even though the algorithm proposed makes sense intuitively and the author provides experimental results with specific data set, I would recommend the author to provide a theoretical justification for the algorithm. For example, it does seem necessary to multiply the temporary mean by the cardinality of the class when calculating the displacement, but it is not clear if this is sufficient to account for the effect that the class with high cardinality eat up every new point due to its gravitational pull (manuscript line 166). The same concern applies to the use of other moments.

2. I recommend the author to include more related methods in the introduction section, such as the nearest centroid classifier and clarify if there is any other similar methods that have been proposed in the literature. This will help to provide a clearer context for the proposed algorithm and its contributions.

3. The algorithm has only been tested on one specific data set, the PID data set. I would recommend the author to test the algorithm on other data sets and compare the performances with other state-of-the-art classification

methods. This will help to validate the effectiveness and generalizability of the proposed algorithm.

Author:

1. To justify the weighting strategy, we have added a whole new section titled "MDEM as a size-weighted minimum perturbation classifier", which establishes MDEM algorithm as a size-weighted minimum perturbation classifier. To justify the weighting strategy of the displacement in moments by the respective class cardinality, here we have given a formal proof of the fact that the displacement in the n-th central moment and displacement in 1st raw moment (mean) of a class due to inclusion of a new instance, are inversely proportional to the respective class cardinality. So, to obtain an unbiased measure of displacement metric, we need to multiply it by the respective class cardinality. The formal proof is given in section 5 of the revised manuscript.

2. Following reviewer's suggestion, we now added a new paragraph regarding Nearest Centroid Classifier (NCC) in our introduction. The paragraph reads as follows.

   "Perhaps one of the most straightforward algorithm for supervised learning is the Nearest Centroid Classifier (NCC) that attempts to estimate the centroid of each class in the training phase, which can be done in $O(n \times d)$, where n is the number of training samples and d is the dimensionality of the feature space [7]. Moreover, in NCC, every new instance can be classified in $O(c \times d)$, where c is the number of centroids, as it involves computing the Euclidean distance between each centroid and the new sample under consideration [8]."

3. Following reviewer's suggestion, we have now replicated the study for Wisconsin breast cancer dataset. Like the PID dataset, we have obtained similar results for Wisconsin breast cancer dataset as well in terms of accuracy, precision, recall, F1 score and MCC.

3.2 Minor comments

Guoning Yu (Reviewer-3)

1. It is better to specify that the algorithm is a supervised learning algorithm for classification in the abstract. Same applies to the introduction.

2. The first sentence of the abstract can be rephrased like: We introduce a supervised learning method that classifies each test point by selecting the class for which its inclusion causes minimum displacement of the class's existing n-th central moment.

3. Manuscript line 36: O(logn) should be O(log n).

4. Every citation mark for the whole manuscript has a space before it, e.g., manuscript line 35, 37, 38, etc.

5. Manuscript line 100: move the commas before both "or"

6. Manuscript line 112-122: remove the colons after all "section", also remove the colons after "Algorithm" and "Fig" in later sections.

7. Manuscript line 142: the attributes should be all numerical according to the context, which is better to be specified.

8. Manuscript line 192: after the training phase, we have the n-th central moment of each class... It should not be mean.

9. Manuscript line 197: should be the number of attributes

Author:

1. Following reviewer's suggestion, we have now explicitly mentioned that we have introduced a new supervised learning algorithm for classification in the abstract. To be precise, we have rewritten the first sentence of the abstract as follows. "We introduce a supervised learning method that classifies each test point by selecting the class for which its inclusion causes minimum displacement of the class's existing n-th central moment."

2. The first sentence in the abstract is replaced according to reviewer's suggestion.

3. Corrected accordingly. O(logn) replaced with O(log n)

4. Spaces are removed accordingly.

5. Both the commas removed accordingly.

6. We have removed colons after every section, algorithm, figure, table and equation.

7. Following reviewer's suggestion, we have now rewritten the line as "Let us also assume that each of the training instances has N number of numerical attributes."

8. "Mean" replaced with n-th central moment accordingly.

9. 'of' added accordingly.

4 Reviewer-4

Reviewer-4

1. All results are from the Pima Indian Diabetes dataset. This limits generalizability. Benchmarking on diverse datasets to stress-test MDEM might be helpful.

2. MDEM's design lacks the flexibility or capacity of nonlinear models like SVM (with kernels) or deep NNs. This inherently caps its accuracy, especially in more complex domains. Would it be possible to combine MDEM with lightweight NNs or tree-based models to handle non-linearity? Kernel tricks may also be able to extend MDEM to non-linear decision boundaries.

3. The intuition behind using minimal moment displacement is compelling but not theoretically grounded. Formal proofs of convergence, error bounds, or probabilistic guarantees are missing.

Author:

1. Following reviewer's suggestion, we have now replicated the whole study for Wisconsin breast cancer dataset. We have reported the new results that are similar to that of PID dataset in our revised manuscript.

2. Reviewer has rightly pointed out that MDEM's design lacks the flexibility or capacity of nonlinear models like SVM (with kernels) or deep NNs and it is evident from the empirical results. We have discussed the issue of sub-optimal performance of MDEM algorithms as compared to other more complex algorithms like SVM and NN. In fact, MDEM algorithms are designed for situations, where speed is preferred to cutting edge accuracy. We have also added a whole new subsection titled "Discussion" under "Results" section of our revised manuscript that addresses the issue. The subsection reads as follows.

   "MDEM algorithms are better suited in numerous situations, where speed in running time is more important than cutting-edge accuracy. For example, MDEM algorithms can be practically deployed to different text classification scenarios, e.g., classifying documents according to search query, email filtering (spam vs non-spam and also categorization and labelling of emails), categorization of news articles (sports, politics, international etc.), populating newsfeeds for users in social media, sentiment analysis (classifying reviews as positive, negative and neutral), support ticket routing (assign incoming tickets to the right department), legal and medical documents tagging (tag documents based on predefined categories) and the alike. These are some of the applications, where speed is preferred to optimality as the data here are massive in quantity and consequences of possible misclassification are relatively less dangerous. "

   However, as the reviewer has rightly mentioned, it is very logical to build some ensemble techniques using MDEM and other relevant linear and non-linear classifiers with a view to improving performance. But, as we have mentioned here, MDEM is designed for speed and coupling it with complex models like SVM and NN with MDEM will inevitably reduce its speed hurting the core use cases of MDEM. That is why, we did not go for it.

3. To address reviewer's concern, we have added a whole new section titled "MDEM as a size-weighted minimum perturbation classifier", which establishes MDEM algorithm as a size-weighted minimum perturbation classifier. To justify the weighting strategy of the displacement in moments by the respective class cardinality, here we have given a formal proof of the fact that the displacement in the n-th central moment and displacement in 1st raw moment (mean) of a class due to inclusion of a new instance, are inversely proportional to the respective class cardinality. So, to obtain an unbiased measure of displacement metric, we need to multiply it by the respective class cardinality. The formal proof is given in section 5 of the revised manuscript.
